# A homozygous missense variant of SUMF1 in the Bedouin population extends the clinical spectrum in ultrarare neonatal multiple sulfatase deficiency

**DOI:** 10.1002/mgg3.1167

**Published:** 2020-02-12

**Authors:** Orna Staretz‐Chacham, Lars Schlotawa, Ohad Wormser, Inbal Golan‐Tripto, Ohad S. Birk, Carlos R. Ferreira, Thomas Dierks, Karthikeyan Radhakrishnan

**Affiliations:** ^1^ Metabolic Clinic Soroka University Medical Center Ben Gurion University Beer Sheva Israel; ^2^ Neonatlogy Unit Soroka University Medical Center Ben Gurion University Beer Sheva Israel; ^3^ Division of Pediatrics Soroka University Medical Center Ben Gurion University Beer Sheva Israel; ^4^ Department of Paediatrics and Adolescent Medicine University Medical Center Goettingen Germany; ^5^ The Morris Kahn Laboratory of Human Genetics National Institute for Biotechnology in the Negev and Faculty of Health Sciences Ben Gurion University of the Negev Beer Sheva Israel; ^6^ Shraga Segal Department of Microbiology, Immunology and Genetics Ben Gurion University of the Negev Beer‐Sheva Israel; ^7^ Genetic Institute Soroka University Medical Center Ben Gurion University Beer Sheva Israel; ^8^ Medical Genomics and Metabolic Genetics Branch National Human Genome Research Institute National Institutes of Health Bethesda MD USA; ^9^ Biochemistry I Faculty of Chemistry Bielefeld University Bielefeld Germany

**Keywords:** deafness, developmental delay, dysmorphism, formylglycine‐generating enzyme, hypotonia, lysosomal storage disorders, Multiple sulfatase deficiency, persistent pulmonary hypertension of the newborn, sulfatases, SUMF1

## Abstract

**Background:**

Multiple sulfatase deficiency (MSD, MIM #272200) is an ultrarare congenital disorder caused by SUMF1 mutation and often misdiagnosed due to its complex clinical presentation. Impeded by a lack of natural history, knowledge gained from individual case studies forms the source for a reliable diagnosis and consultation of patients and parents.

**Methods:**

We collected clinical records as well as genetic and metabolic test results from two MSD patients. The functional properties of a novel SUMF1 variant were analyzed after expression in a cell culture model.

**Results:**

We report on two MSD patients—the first neonatal type reported in Israel—both presenting with this most severe manifestation of MSD. Our patients showed uniform clinical symptoms with persistent pulmonary hypertension, hypotonia, and dysmorphism at birth. Both patients were homozygous for the same novel SUMF1 mutation (c.1043C>T, p.A348V). Functional analysis revealed that the SUMF1‐encoded variant of formylglycine‐generating enzyme is highly instable and lacks catalytic function.

**Conclusion:**

The obtained results confirm genotype‒phenotype correlation in MSD, expand the spectrum of clinical presentation and are relevant for diagnosis including the extremely rare neonatal severe type of MSD.

## Graphical abstract

Multiple sulfatase deficiency (MSD) is an ultra‐rare congenital disorder caused by SUMF1 dysfunction and often misdiagnosed due to its complex clinical presentation. Here we report two Israeli patients with a neonatal MSD caused by a likely‐founder mutation SUMF1‐A348V, in the Bedouin population. We show that A348V mutation leads to loss of stability of SUMF1 resulting in loss of function. The presented clinical and biochemical findings expand the spectrum of MSD, adding data on the rare neonatal severe type of MSD.

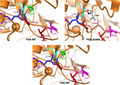

## 1 INTRODUCTION

Multiple sulfatase deficiency (MSD; MIM #272200) is a rare, yet untreatable lysosomal storage disorder inherited in an autosomal recessive manner. MSD leads to deficiency of all sulfatases, such as those associated with Hunter syndrome, Morquio syndrome, Maroteaux‒Lamy syndrome, metachromatic leukodystrophy, X‐linked ichthyosis, and X‐linked chondrodysplasia punctata (Hopwood & Ballabio, 2001). MSD is caused by mutations in the sulfatase‐modifying factor 1 gene (*SUMF1*) located on chromosome 3p26, which encodes the formylglycine‐generating enzyme (FGE). FGE posttranslationally activates all 17 human sulfatases (Cosma et al., 2003; Dierks, Schmidt, & Borissenko, 2003) by generating the catalytic formylglycine residue in the sulfatases active site. Most sulfatases are responsible for the breakdown and recycling of complex sulfate‐containing molecules and therefore mutations in the *SUMF1* result in tissue accumulation of sulfated glycosaminoglycans, sulfatides, and also steroid sulfates (Dierks, Dickmanns, & Preusser‐Kunze, 2005; Dierks et al., 2009). At least nine different single sulfatase deficiencies are known to be associated with a specific congenital disease, most of which are lysosomal storage disorders (Dierks et al., 2009; Khateb, Kowalewski, & Bedoni, 2018). The combined deficiency of sulfatases results in a clinical phenotype comprising a variable combination of features of single sulfatase deficiencies in each MSD patient (Ahrens‐Nicklas, Schlotawa, & Ballabio, 2018). There are three clinical types of the disease according to the age of onset and disease severity: (a) neonatal, the most severe type which results in death within the first year in most reported cases; (b) late infantile, the most common one; and (c) juvenile type (Eto, Gombuchi, Umezawa, & Tsuda, 1987; Sabourdy, Mourey, & Le Trionnaire, 2015; Schlotawa, Ennemann, & Radhakrishnan, 2011). A genotype‒phenotype correlation for MSD has been proposed based on experimental data and correlation with the clinical course of MSD patients: SUMF1 mutations severely affecting protein stability and enzymatic function result in more severe forms of MSD, whereas mutations allowing residual enzymatic activity and protein stability were found in attenuated cases (Jaszczuk, Schlotawa, & Dierks, 2017; Sabourdy et al., 2015; Schlotawa et al., 2011). So far, less than 100 MSD cases with any type have been reported worldwide. No comprehensive natural history data on MSD have been published yet. Clinical knowledge still relies on the description of individual cases thereby adding to the disease spectrum.

We present two new cases of MSD with the neonatal type in the Bedouin community. Both are homozygous for the same novel variant in *SUMF1*, and share similar symptoms from birth, thereby extending the clinical phenotype of neonatal MSD.

## 2 METHODS

### 2.1 Patient consent and institutional review board approval

The study was approved by the local institutional review board. Informed consent, was obtained from the parents on behalf of each child.

### 2.2 Lysosomal hydrolase activity assays

**Figure 1:**
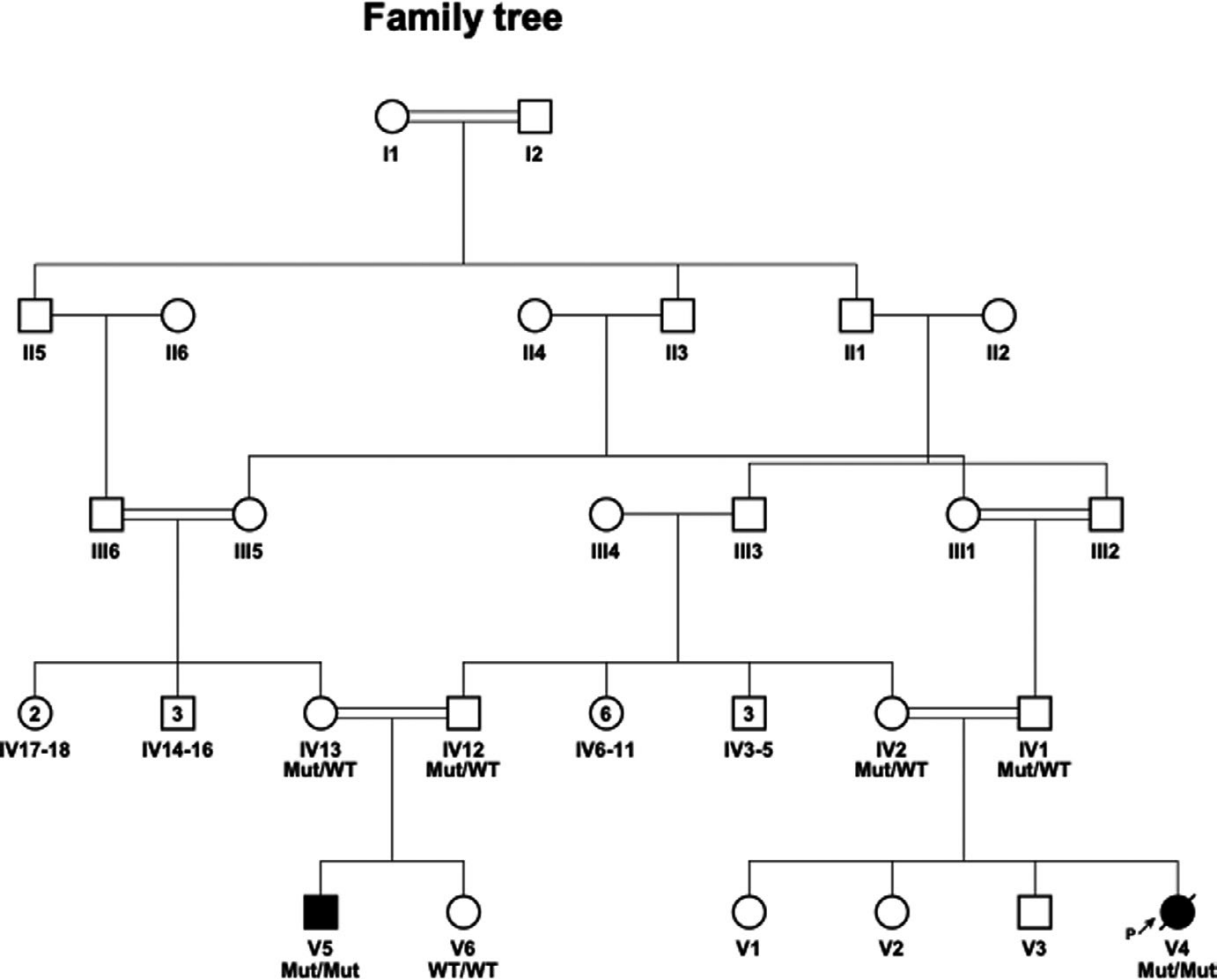
Pedigree. Family tree of the extended family. Probands are V4 and V5

Peripheral blood samples were collected from the patients and parents, and DNA was extracted from blood samples according to standard protocols (Centogene). Blood spots of the proband V4 (Figure 1) were collected on filter card and dried as in standard protocols. Initially, the proband's sample was sent for enzyme activity analysis of alpha‐iduronidase, galactosamine‐6‐sulfate sulfatase, beta‐galactosidase, arylsulfatase B, and iduronate‐2‐sulfatase to Centogene (Rostock, Germany). Arylsulfatase B and iduronate‐2‐sulfatase activities were measured in dried blood spots by fluorometry (based on the fluorometric determination of 4‐methylumbelliferone) while the enzyme activities of alpha‐iduronidase, beta‐galactosidase, galactosamine‐6‐sulfate sulfatase, and acidic sphingomyelinase (the latter as a control) were measured by tandem mass spectrometry (Centogene).

### 2.3 Molecular genetics and sequencing

The *ARSB*, *GALNS*, *IDS*, and *SUMF1* were analyzed by PCR and Sanger sequencing for the proband (patient V4), performed by Centogene (Rostock, Germany). The four genes’ entire coding region (including all major transcripts: NM_000046.3, NM_198709.2, NM_000512.4, NM_001323544.1, NM_000202.5, NM_006123.4, NM_182760.3) and the highly conserved exon‒intron splice junctions were sequenced. Quantitative PCR (qPCR) assay was performed using gene‐specific amplicons encompassing every coding exon (or part of it) of the *GALNS* (NM_000512.4). Sanger sequencing was used to perform segregation analysis of the *SUMF1* variant detected in the studied Bedouin kindred.

### 2.4 cDNA and expression constructs

The generation of pBI expression constructs encoding FGE‐HA and steroid sulfatase (STS) was done as described earlier (Mariappan, Gande, et al., 2008). Briefly, the cDNA encoding human FGE with a C‐terminal HA tag was cloned into the MCS‐II to generate pBI‐FGE‐HA and human STS cDNA was cloned into MCS‐I of pBI vector or pBI‐FGE‐HA to generate pBI‐STS or pBI‐FGE‐HA+STS plasmid, respectively, thus allowing expression of both the proteins under a doxycycline‐inducible bi‐directional promotor. FGE‐A348V mutation was created by site‐directed mutagenesis PCR with pBI‐FGE‐HA or pBI‐FGE‐HA+STS plasmid as template and complementary primers (FGE‐A348V_Fwd, 5′‐AGGTATCGCTGTGCTGTTCGGAGCCAGAACACA‐3′ and FGE‐A348V_Rev, 5′‐TGTGTTCTGGCTCCGAACAGCACAGCGATACCT‐3′). The cDNA encoding human ARSA with a C‐terminal RGS‐His tag was cloned either into the MCS‐I of pBI vector or pBI‐FGE‐HA to generate pBI‐ARSA‐His or pBI‐FGE‐HA+ARSA‐His plasmid, respectively. All expression plasmids were verified by sequencing of the entire coding region to exclude any undesired PCR‐derived errors.

### 2.5 Cell culture and transfection

The generation of stable TetOn cell lines of HT1080 fibrosarcoma cells and immortalized MSD (MSDi) patient skin fibroblasts (a gift from Andrea Ballabio's lab, Italy) were as described earlier (Mariappan, Gande, et al., 2008). The cells were maintained under 5% CO_2_ at 37°C in Dulbecco's modified Eagle's medium supplemented with 10% fetal calf serum, 1% penicillin/streptomycin (Invitrogen) and 800 µg/ml G418 (HT1080‐TetOn cells) and 5 µg/ml blasticidin (MSDi‐ TetOn cells). Transfection of plasmids was performed with PEI and 4 to 6‐hr posttransfection, protein expression was induced with medium containing 2 µg/ml doxycycline for 22–24 hr after which the cells and medium were harvested.

### 2.6 Sulfatase activity assays and western blotting

The STS activity assay in MSDi cells was performed as described earlier (Mariappan, Gande, et al., 2008). Briefly, the activity was performed in cell lysates that transiently express either STS alone or STS together with FGE. ARSA activity assay was performed in MSDi cell lysates that transiently express either ARSA‐His alone or ARSA‐His together with FGE. ARSA activity toward pNCS (*para*‐nitrocatechol sulfate) as substrate was carried out with 10 mM pNCS in 0.5 M sodium acetate buffer (pH 5.5) containing 10% (w/v) NaCl and 2 mM sodium pyrophosphate. The cell lysates were incubated at 37°C for 1.5 to 4 hr in a total 250 µl reaction volume. The reactions were stopped by addition of 200 µl 1 M NaOH. Absorbance were measured at 515 nM using an Infinite M200 microplate reader (TECAN, Crailsheim) and activity was calculated using the molar extinction coefficient (e_515_ = 12,400 M^−1^ cm^−1^) of pNCS. The specific activity of respective sulfatases was calculated by dividing the activity in lysates by the western blot signal (arbitrary units).

For western blot analyses, 50 µg of total protein was resolved by SDS‐PAGE and proteins were transferred to PVDF membrane followed by incubation with respective antibodies. Rabbit polyclonal antisera against FGE and STS or mouse monoclonal anti‐RGS‐His antibody for ARSA‐His were used as primary antibodies and HRP‐conjugated goat anti‐rabbit antibodies or goat anti‐mouse antibodies were used as secondary antibodies. Western blot signals were quantified using the AIDA 2.1 software (Raytest).

### 2.7 Intracellular stability analysis by cycloheximide chase assay

HT1080‐TetOn cells grown in 3 cm plates were transiently transfected with either pBI‐FGE‐WT‐HA or pBI‐FGE‐A348V‐HA using PEI as transfection reagent. 4‒6‐hr posttransfection, the protein expression was induced by addition of 0.5 µg/ml doxycycline. After 2.5 hr of protein expression, the doxycycline containing medium was removed, cells washed twice with 1X PBS to remove excess doxycycline. The cells were harvested immediately (0h) or incubated further with 1 ml media containing 250 µg/ml cycloheximide. At timepoints 2, 4, and 6 hr the cells and media were harvested. The cells were harvested by scrapping in 200 µl of ice‐cold lysis buffer (1× PBS + 1:100 protease inhibitor cocktail) and lysed by sonication on ice for 3 × 20 s. Secreted FGE was immunoprecipitated from the medium by incubation with rabbit FGE anti‐serum overnight at 4°C, followed by incubation with Pansorbin (Calbiochem) at room temperature for 1 hr. The pansorbin pellet containing immunoprecipitated FGE was washed four times with 1× PBS containing 0.25% Triton X‐100 and once with 1× PBS. The bound FGE was eluted by boiling the pellet in 1× Laemmli buffer at 95°C for 5 minutes. To analyze the amount of FGE in the cells and medium, 50 µg of the cell lysate and immunoprecipitated FGE from the medium were resolved by SDS‐PAGE and proteins transferred to PVDF membrane by western blotting. The membrane was probed with rat monoclonal anti‐Hsc70 (as loading control) and mouse monoclonal anti‐HA antibodies. The western blot signals were quantified using AIDA 2.1 software (Raytest). After normalizing the signals corresponding to FGE‐HA in the cell lysates to Hsc70, the amount of FGE in both the cells and medium per mg of total protein was compared.

## 3 RESULTS

### 3.1 Clinical studies

**Family A, patient/V4** (Figure 1) is the fourth child of consanguineous Bedouin parents. She was born at term, following a prenatal diagnosis of muscular ventricular septal defect (VSD) by fetal echocardiogram, and chorionic villous sampling that ruled out homozygosity for various tribal founder mutations. Birth weight was 3,580 grams and head circumference 33.5 cm (<90th and <50th pecentile, respectively). She presented at birth with dysmorphism (long eyelashes, low anterior hairline, low‐set ears), fisting with persistent adduction of thumbs, hypotonia of lower limbs > upper limbs, locked knees and decreased primitive reflexes, no rooting and only partial suck, pulmonary hypertension, failed hearing screen, and jaundice. Pulmonary hypertension necessitated oxygen support by nasal cannula for 5 days.

The patient had recurrent admissions starting at 4 months of age due to worsening reactive airway disease that required noninvasive ventilation with Continuous Positive Airway Pressure (CPAP) and oxygen support. Bronchoscopy at 10 months of age revealed large nasopharyngeal hypertrophic adenoid and mild laryngomalacia. During the second year of life she developed dry skin that got worse over time.

Developmental delay was noted from birth. She started rolling at 8 months of age and also started babbling but did not reach other milestones. At 4 months of age she developed upbeat nystagmus. Her development was completely arrested at 18 months, followed by regression. Brain MRI at 12 months disclosed partial corpus callosum agenesis, mild external hydrocephalus, and suspected facial nerve aplasia. Electroencephalogram (EEG) performed at 34 months revealed depressed background activity with no seizures.

As part of her workup she underwent a brainstem auditory evoked response (BEAR) test, which revealed bilateral severe sensorineural hearing loss. She had cochlear implants with adenoidectomy and frenulectomy at 18 months. Eye exam at 30 months showed diffuse stromal corneal clouding. She continued to deteriorate and at 34 months had a gastric tube insertion. Recurrent admissions for respiratory support due to aspirations became necessary. She died at the age of 38 months due to respiratory failure. Laboratory investigations due to suspicion of mucopolysaccharidosis included urinary glycosaminoglycans (GAGs) at 10 months (275 mg/mmol creatinine; control < 17 mg/mmol creatinine) followed by enzymatic workup (see Table 1).

**Table 1:** Biochemical features of patient A‐V4

	Patient A‐V4	
Enzyme	Measured activity µmol/l/h	Reference µmol/l/h
Galactosamine−6‐sulfate sulfatase	**<0.3 (LOQ)**	**≥2.0**
Arylsulfatase B	**1.4**	**≥8.8**
Iduronate−2‐sulfatase	**<2.8 (LOQ)**	**≥5.6**
α‐Iduronidase	23.2	≥2.9
ß‐Galactosidase	136.4	≥28.5

Note

**Bold**‐ pathologic values.

Abbreviation: LOQ, limit of quantitation.

**Family B, Patient/V5** (Figure 1) is the first son in family B. He was born at term with a birth weight of 4,265 grams and head circumference of 37 cm (>90% percentile). Prenatal genetic workup for known family founder mutations was negative. Fetal ultrasound revealed left clubfoot, while fetal MRI and echocardiogram were normal. At birth, he needed resuscitation with suspected neonatal encephalopathy due to low cord pH. He also presented with marked dysmorphism (coarse facial features with low‐set ears) at birth and additional bilateral broad thumbs and bilateral clubfeet, undescended testes and inguinal hernia, hypotonia, and pulmonary hypertension that required oxygen supply. The patient also presented congenital thrombocytopenia that resolved spontaneously with no apparent etiology, as well as mild hepatosplenomegaly. An echocardiogram at 4 weeks of age was normal, at 12 months of age mild aortic regurgitation was noted.

**Figure 2:**
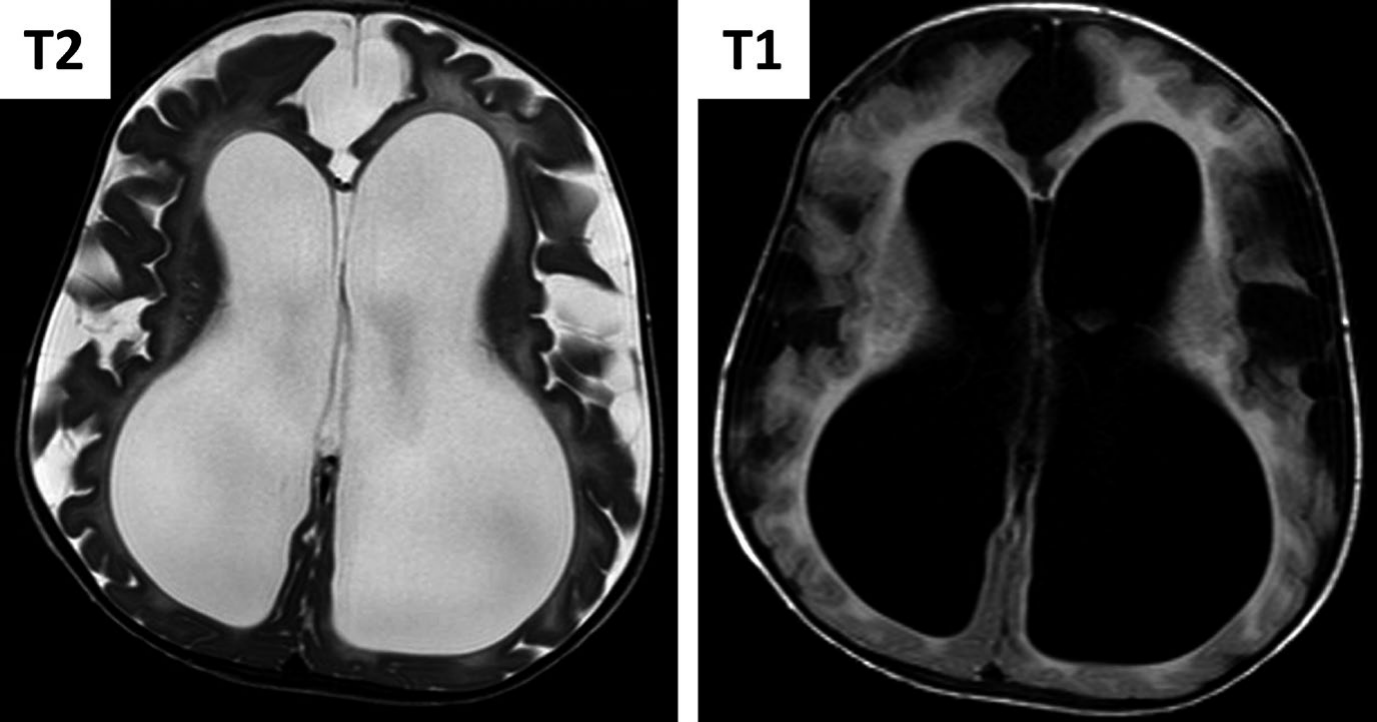
Brain MRI of patient V5 at 21 months. T1 (Right) and T2 (Left) weighted MRI showing white and gray matter atrophy with secondary enlarged lateral ventricles

The patient had recurrent admissions beginning at 8 weeks of life due to worsening reactive airway disease, with development of severe obstructive sleep apnea at 31 months that necessitated continuous CPAP support with oxygen. During the second year of life he developed worsening dry skin that developed into ichthyosis over time. Developmental delay was noted from birth. He started rolling at 8 months and in addition started babbling and reaching for objects at the same time, but did not reach other milestones, such as sitting, crawling or walking. His development completely arrested and regressed at 21 months. He developed increasing irritability. A cerebral MRI at 21 months revealed severe hydrocephalus, which necessitated an insertion of a ventriculoperitoneal shunt (Figure 2). BAER test disclosed mild left ear sensorineural hearing loss. Eye exam at 28 months showed diffuse stromal corneal clouding.

Laboratory investigations due to suspicion of mucopolysaccharidosis included urinary GAGs at 10 months (215 mg/mmol creatinine; control < 36 mg/mmol creatinine) followed by enzymatic workup—alpha‐iduronidase(18.1 nmol/spot*20 hr; Control:400–3300 nmol/spot*20 hr), beta‐galactosidase (0.52 nmol/spot*20 hr; Control:0.5–3.2 nmol/spot*20 hr), and arylsulfatase B (0.09 nmol/spot*20 hr; Control:0.15–0.9 nmol/spot*20 hr). Molecular diagnosis was obtained at 8 months of age and the parents were found to be heterozygous for the SUMF1 mutation. The patient is now 2 years and 10 months old and continues to deteriorate.

### 3.2 Genetic analysis

Sanger sequencing of patient/V4 detected no pathogenic variants in the *IDS*, *GALNS,* and *ARSB*. We could also exclude multiexonic or whole‐gene deletions/duplications within or encompassing the *GALNS*. Enzymatic activity testing showed decreased activities for three sulfatases tested (Table 1).

Sequencing of the SUMF1 gene reveled a previously unreported homozygous variant in exon 9, (c.1043C>T, p.A348V, NM_182760). Polyphen‐2 (Pearl, Causality, Kalisch, Bühlmann, & Hughes, 2010), SIFT (Sim et al., 2012), and MutationTaster (Schwarz, Cooper, Schuelke, & Seelow, 2014) predicted this variant to be likely damaging. This variant is not described in the Exome Aggregation Consortium (ExAC) or in the genome aggregation database (gnomAD). Furthermore, this variant was not found in our in‐house whole‐exome dataset of 236 ethnically matched individuals. The variant segregated as expected for an autosomal recessive trait in the pedigree (Figure 1) and was found in a homozygous state also in affected patient/V5.

### 3.3 Functional consequences of the patient mutation on FGE activity and stability

**Figure 3:**
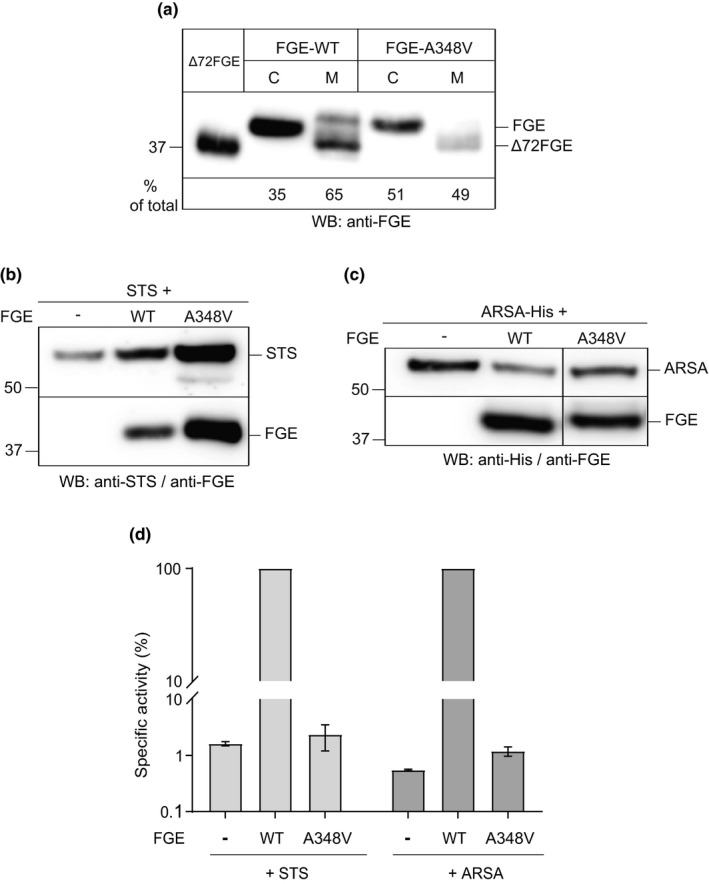
Intracellular retention and activity of FGE‐A348V. (a) HT1080‐TetOn cells were transiently transfected with plasmids encoding either FGE‐WT or FGE‐A348V and expression was induced with 2 µg/ml doxycycline for 24 hr. Cell lysate (C) and medium (M) in a ratio of 4:1 were resolved in SDS‐PAGE and western blot probed with FGE‐antiserum. The signals corresponding to FGE in western blot were quantified using a known amount (100 ng) of purified Δ72FGE loaded as standard. The amount of FGE (mg/ml) in cells and medium was calculated and expressed as percentage of total FGE. b, c, d) MSDi‐TetOn cells (in triplicates) were transiently transfected with plasmids encoding either STS alone or STS + FGE (b) and either ARSA alone or ARSA + FGE (c). After 24 hr of doxycycline‐induced expression, the activity of sulfatases was measured in the respective cell lysates and specific activities were calculated with amounts of sulfatases quantified from western blots shown in c (representative of three independent experiments). The bar diagram (d) depicts the comparison of the relative specific activity of STS and ARSA in cells, that is, in percentage relative to activity of FGE‐WT coexpressing cells (100%). The values represent mean ± *SEM* of three independent experiments

Being an uncharacterized novel mutation, we first assessed the conservation of residue Ala348. Analysis of FGE sequences from 29 different species of higher mammals revealed that Ala348 is part of a stretch of residues, which are highly conserved with a 100% conservation of alanine at this position (Figure S1). We next sought to analyze the biochemical characteristics of this variant. FGE is a secreted protein but also retained in the endoplasmic reticulum (ER) and a major fraction of FGE is secreted as a N‐terminally truncated form (Δ72FGE) due to furin processing (Ennemann, Radhakrishnan, & Mariappan, 2013; Mariappan, Radhakrishnan, Dierks, Schmidt, & Figura, 2008). Since it is known that most of the disease‐causing missense mutations in FGE lead to its misfolding‐mediated ER‐retention we, as a first step, analyzed the secretion properties of the FGE‐A348V variant in comparison to wildtype (FGE‐WT). Western blot analysis of cell lysates and media from cells transiently expressing either FGE‐WT or its A348V variant showed that, compared to WT, about 1.5‐fold more of the variant is retained intracellularly indicating an altered secretion behavior probably due to failure in passing the ER quality control system (Figure 3a).

To gain insights into the consequence of A348V exchange on the intracellular activity of FGE, we analyzed FGE‐mediated activation of steroid sulfatase (STS) and arylsulfatase A (ARSA) using standardized in vivo sulfatase activity assays in immortalized MSD (MSDi) cells (Mariappan, Gande, et al., 2008). Sulfatase activity assays were performed either in MSDi cell lysates that express the sulfatases alone or along with FGE‐WT or FGE‐A348V (Figure 3b,c). Coexpression of FGE‐WT led to a 75‐fold and 150‐fold increase in the activity of STS and ARSA, respectively (data not shown). However, coexpression of FGE‐A348V led to no further increase in the activity of either STS or ARSA above the background activity observed in cells expressing either of these sulfatases alone, that corresponds to only around 1% of activity compared to FGE‐WT (Figure 3d). In conclusion, the A348V mutation completely abolishes the in vivo activity of FGE toward the two sulfatases tested.

**Figure 4:**
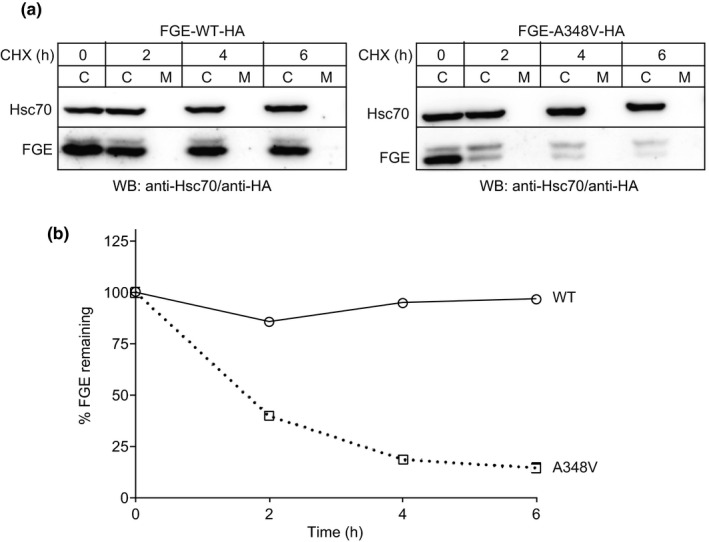
Intracellular stability of FGE‐A348V. (a) HT1080‐TetOn cells were transiently transfected with plasmids encoding either FGE‐WT‐HA (left panel) or FGE‐A348V‐HA (right panel). After 2.5 hr of protein expression (induced with 0.5 µg/ml doxycycline) the cells were subjected to cycloheximide chase analysis for 6 hr with cells and media collected at the indicated timepoints. Cell lysates and FGE‐antiserum‐immunoprecipitates from media (see experimental procedures) were resolved in SDS‐PAGE and western blot decorated with anti‐HA (to detect FGE) and anti‐Hsc70 (as loading control). (b) Plot depicting the percentage of FGE remaining after quantification of data in (a) using AIDA 2.1 software. After normalization of the anti‐HA antibody signals (corresponding to FGE) in cell lysates to anti‐Hsc70 signals, the total amount of FGE (µg/mg of total protein in lysate) in the cells and media was combined and expressed as the percentage of that at the start of the chase (0 hr)

We then analyzed the effect of the A348V mutation on the intracellular stability of FGE using cycloheximide chase analysis (Figure 4). After induction of protein expression for 2.5 hr in HT1080 cells, transiently expressing either FGE‐WT or FGE‐A348V, the amount of FGE in the cells and media was determined after cycloheximide treatment by western blot analysis for up to 6 hr (Figure 4a). A decrease in the signals for intracellular FGE over time was clearly observable for the A348V variant, whereas the FGE‐WT level had been stable. Quantification of the western blot signals revealed no change in the levels of FGE‐WT over the analyzed time, whereas a 90% decrease in the total levels of A348V in 6h, with a drastic decrease of more than 50% within the first 2 hr, suggests a very low intracellular stability (Figure 4b). Based on the known 3D structure of human FGE, analysis of the effect of the A348V mutation by in‐silico mutagenesis revealed that clashes of the bulky sidechain of valine in particular with the neighboring sidechain of Arginine345 and the ensuing loss of the integrity of the active site region could be the basis for misfolding and reduced intracellular stability (Figure S2).

## 4 DISCUSSION

Neonatal presentation in MSD is considered to be the rarest and most severe type. Only six descriptions of the neonatal type have been published. However, symptoms at birth could have been missed in other severe or neonatal MSD cases and could have resulted in an underestimation of the diagnosis. Both cases reported in our study presented with pulmonary hypertension, muscular hypotonia, and dysmorphic features leading to a suspicion of mucopolysaccharidosis and were tested accordingly. The diagnosis in the first patient was only suspected at 8 months of age despite the presence of symptoms at birth. Apart from a report on a patient from Israel affected with an intermediate severe late infantile form of MSD (Zilberman & Bibi, 2016), our study is the first report of neonatal type MSD in Israel.

As in previous reports of neonatal MSD cases dysmorphic features were one of the leading clinical symptoms resulting in a clinical workup for suspicion of MPS disorders. Dysmorphic, MPS‐like appearance in MSD appears to be frequently described as first symptom in neonatal and severe cases. By contrast, MSD cases of the late infantile type, which accounts for the majority of cases, were described to present with dysmorphic features only later in life. However, it cannot be ruled out that subtle changes were missed or not described in such patients (Ahrens‐Nicklas et al., 2018). Apart from dysmorphism our patients display clinical symptoms that fit very well with other patients presenting with neonatal MSD (see Table 2). In contrast to previous reports, our patients did not show intrauterine growth retardation, but developed failure to thrive over time (Burch, Fensom, Jackson, Pitts‐Tucker, & Congdon, 1986; Busche, Hennermann, & Bürger, 2009; Vamos, Liebaers, Bousard, Libert, & Perlmutter, 1981). Ichthyosis was thought to be an important indicator of MSD and the best marker of the disease in neonates (Garavelli, Santoro, & Iori, 2014). However, none of our patients had ichthyosis at birth but developed dry skin and ichthyosis later. Both patients showed clear signs of airway involvement, including pulmonary hypertension, hyper‐reactive airway disease with frequent exacerbations and the second patient presented with obstructive sleep apnea, both symptoms not frequently described in MSD but of clinical importance.

**Table 2:** Clinical features of our two patients diagnosed with MSD in comparison to other published neonatal MSD cases

Clinical feature	Patient A‐V4	Patient B‐V5	Vamos et al. (1981)	Burch et al. (1986)	Busche et al. (2009)	Schlotawa et al. (2011)	Garavelli et al. (2014)	Güzel Nur et al. (2014)
Prenatal findings	VSD	Lt. club foot	Placental insufficiency	n.d	Fetal ascites/ hydrops	n.d	n.d	Congenital ascites and oligohydramnios
Muscular hypotonia	+	+	+	+	+	n.d	+	+
Developmental delay	+	+	+	n.d	+	+	+	+
Seizures	−	−	−	−	−	−	+	+
Dysmorphism	+	+	+	+	+	+	+	+
Cloudy cornea	+	+	+	+	−	+	−	+
Neurologic regression/arrest	+	+	n.d	n.d	n.d	+	+	+
Cardiac involvement	+	+	−	+	+	+	−	+
Organomegaly	+	+	+	+	+	+	+	+
Nephrocalcinosis	−	−	−	−	−	−	−	+
Skeletal changes	+	+	+	+	+	n.d	+	+
Hydrocephalus	+*	+	+	+	+	+	+	+
OSA	−	+	−	+	+	n.d	+	−
Reactive airway disease	+	+	−	−	+	n.d	−	−
Dry skin/Ichthyosis	+	+	+	n.d	+	+	+	−
Deafness/hypoaccousis	+	+	n.d	n.d	+	n.d	n.d	+

Abbreviations: n.d., not determined; OSA, Obstructive Sleep Apnea.

* External Hydrocephalus.

Neonatal MSD was previously reported to result in early death, with some patients not reaching the second year of life (Burch et al., 1986; Busche et al., 2009; Perlmutter‐Cremer, Libert, Vamos, Spehl, & Liebaers, 1981; Schlotawa et al., 2011; Vamos et al., 1981). Despite their severe involvement, our patients survived beyond 2 years of age. Advances in treatment and care of critically ill patients might have an impact on the survival of MSD patients, especially the neonatal cases, and prolong their lifespan. The substantial variability of clinical presentation in MSD was also reflected in our patients carrying the same homozygous pathogenic SUMF1 mutation.

We found a yet undescribed *SUMF1* missense mutation resulting in an A348V substitution in the FGE protein. The same A348 FGE residue was found to be affected in a compound heterozygous patient with moderate‒mild phenotype carrying an exchange to proline (p.A348P) in combination with another missense mutation (p.M1V) on the second allele (Cosma et al., 2003). The milder phenotype in this patient can be explained by our finding that the p.M1V‐variant is expressed, albeit at low levels, and hence is no null mutation (data not shown). Clearly, our functional analysis of the p.A348V variant revealed that the specific substitution of this highly conserved A348 residue dramatically impairs protein function and stability classifying p.A348V as a severe mutation. Our findings corroborate the previously established indication that disease severity in MSD is determined by both protein stability and residual activity (Sabourdy et al., 2015; Schlotawa et al., 2011; Schlotawa, Steinfeld, Figura, Dierks, & Gärtner, 2008). For the two patients described here, the clinical presentation together with our biochemical data provide evidence that the drastic reduction in intracellular FGE stability is the major defining cause resulting in the loss of FGE activity and a neonatal manifestation of MSD.

We assume that the neonatal presentation overlaps with other MSD phenotypes and represents the severe end of a disease spectrum rather than a separate disease entity. The difficulties and a delay in establishing the correct diagnosis might account for a misleading classification. We encourage to include the differential diagnosis of MSD in any clinical workup for a suspicion of a lysosomal storage disorder in newborns. We strongly recommend to measure glycosaminoglycan and sulfatide excretion and at least two individual sulfatase activities followed by molecular genetics of SUMF1 in order not to underdiagnose MSD.

## CONFLICT OF INTEREST

The authors declare that they have no conflict of interest.

## AUTHORS' CONTRIBUTIONS

OSC was responsible for planning, conducting, and reporting of the study. KR and LS were responsible for the functional studies and wrote and revised the manuscript. TD revised the functional data and the manuscript. IGT and CF were part of planning and revising the manuscript. OW and OB were responsible for the genetic studies.
