# Supplementary material for: A homozygous missense variant of SUMF1 in the Bedouin population extends the clinical spectrum in ultrarare neonatal multiple sulfatase deficiency
Source: Mol Genet Genomic Med. 2020 Feb 12;8(9):e1167. doi: 10.1002/mgg3.1167 (PMC7507568; doi:10.1002/mgg3.1167)
Supplement: Supplementary file 1 [file MGG3-8-e1167-s001.pdf]

Supplementary Data

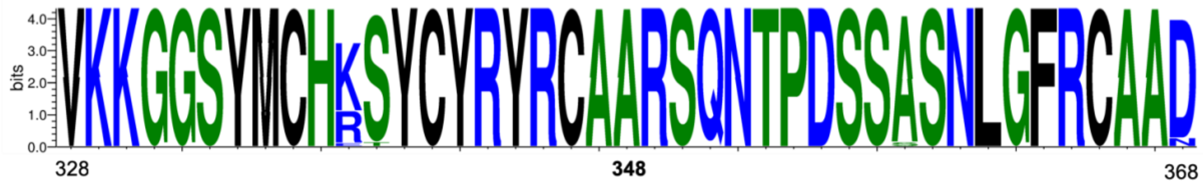

Fig. S1. Conservation of alanine 348 in FGE

The sequence logo for the conservation of A348 was generated using the WebLogo 3.0 program. FGE protein sequences (corresponding to residues 328-368 of human FGE) from various higher mammalian species comprising Euarchontoglires, Laurasiatheres and Atlantogenata were aligned using ClustalW and used for analysis. The sequences from 29 species that were used for the logo generation are given in Table S1.

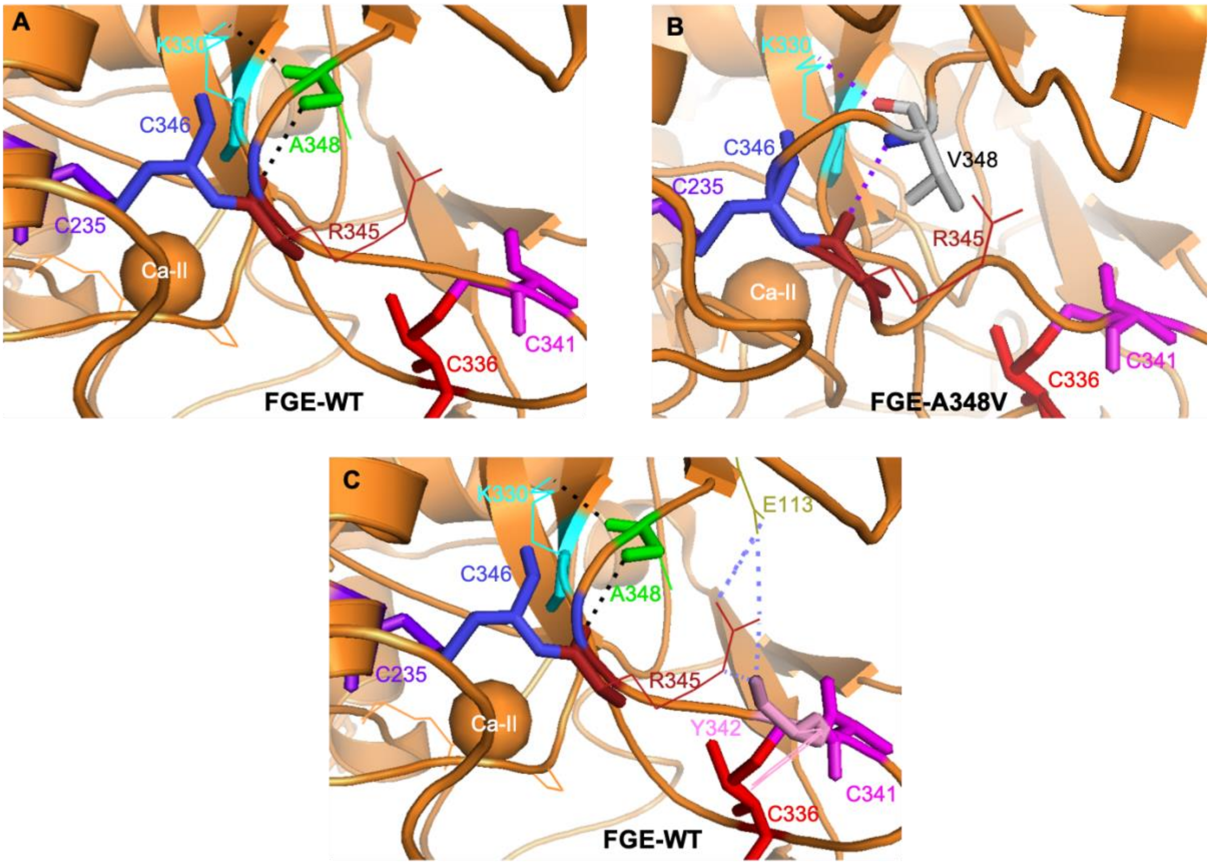

Fig. S2. Effect of FGE-A348V mutation on FGE structure (*in silico* mutagenesis)

Based on the X-ray coordinates of human FGE crystal structure (PDB:1Y1E), the polar contacts between residues were analysed and in-silico mutagenesis was computed using PyMOL. The residues of interest are highlighted and depicted with main chain as sticks and side chains as lines. The interactions between residues are shown in dotted lines. A) Interactions of residue Alanine-348 (A348), B) mutagenesis of A348 to valine (V348), C) interactions of residue Arginine-345 (R345).

Note that the main chain of A348 is involved in interactions with the main chains of R345 and Lys330 (K330) (Fig. S2A). These interactions were still maintained after exchanging A348 with valine by *in-silico* mutagenesis (Fig. S2B). However, the bulky side chain of the introduced valine showed clashes with the side-chain of R345. The side chain of R345 is involved in interaction with side chain of Glu113 (E113) and divalent interactions with the side chain of Tyr342 (Y342) (Fig. S2C). These crucial interactions of R345 could be disturbed by the clashing side chain of valine affecting, in particular, interactions with Y342 which is a part of the catalytic site and the substrate-binding groove. It can be expected that the A348V substitution imposes a loss of the integrity of the residues that stabilize the structure around the active site.

Table S1. FGE sequences used for the WebLogo generation in Fig. S1.

Species	Classification	Sequence	No. of sequences
Homo sapiens		VKKGGSYMCHRSYCYRYRCAARSQNTPDSSASNLGFRCAAD	
Pan troglodytes		VKKGGSYMCHRSYCYRYRCAARSQNTPDSSASNLGFRCAAN	
Pongo abelii		VKKGGSYMCHRSYCYRYRCAARSQNTPDSSASNLGFRCAAD	
Nomascus leucogenys		VKKGGSYMCHRSYCYRYRCAARSQNTPDSSASNLGFRCAAD	
Macaca mulatta		VKKGGSYMCHRSYCYRYRCAARSQNTPDSSASNLGFRCAAD	
Papio anubis		VKKGGSYMCHRSYCYRYRCAARSQNTPDSSASNLGFRCAAD	
Callithrix jacchus		VKKGGSYMCHRSYCYRYRCAARSQNTPDSSASNLGFRCAAD	
Carlito syrichta		VKKGGSYMCHRSYCYRYRCAARSQNTPDSSASNLGFRCAAD	
Otolemur garnettii	Euarchontoglires	VKKGGSYMCHRSYCYRYRCAARSQNTPDSSSSNLGFRCAAD	
Tupaia chinensis		VKKGGSYMCHKSYCYRYRCAARSQNTPDSSASNLGFRCAAD	
Mus musculus		VKKGGSYMCHKSYCYRYRCAARSQNTPDSSASNLGFRCAAD	
Rattus norvegicus		VKKGGSYMCHKSYCYRYRCAARSQNTPDSSASNLGFRCAAD	
Ictidomys tridecemlineatus		VKKGGSYMCHKTYCYRYRCAARSQNTPDSSASNLGFRCAAD	
Dipodomys ordii		VKKGGSYMCHKSYCYRYRCAARSQNTPDSSASNLGFRCAAD	
Oryctolagus cuniculus		VKKGGSYMCHKSYCYRYRCAARSQNTPDSSASNLGFRCAAD	29
Ochotona princeps		VKKGGSYMCHKSYCYRYRCAARSQNTPDSSASNLGFRCAAD	
Canis lupus familiaris		VKKGGSYMCHKSYCYRYRCAARSQNTPDSSASNLGFRCAAD	
Felis catus		VKKGGSYMCHKSYCYRYRCAARSQNTPDSSASNLGFRCAAD	
Ursus maritimus		VKKGGSYMCHKSYCYRYRCAARSQNTPDSSASNLGFRCAAD	
Ailuropoda melanoleuca		VKKGGSYMCHKSYCYRYRCAARSQNTPDSSASNLGFRCAAD	
Pteropus vampyrus	Laurasiatheres	VKKGGSYMCHESYCYRYRCAARSQNTPDSSASNLGFRCAAD	
Sus scrofa		VKKGGSYMCHKSYCYRYRCAARSQNTPDSSASNLGFRCAAD	
Equus caballus		VKKGGSYMCHKSYCYRYRCAARSQNTPDSSASNLGFRCAAD	
Bos taurus		VKKGGSYMCHKSYCYRYRCAARSQNTPDSSASNLGFRCAAD	
Capra hircus		VKKGGSYMCHKSYCYRYRCAARSQNTPDSSASNLGFRCAAD	
Ovis aries		VKKGGSYMCHKSYCYRYRCAARSQNTPDSSASNLGFRCAAD	
Erinaceus europaeus		VKKGGSYMCHKSYCYRYRCAARSQNTPDSSASNLGFRCAAD	
Loxodonta africana	Atlantogenata	VKKGGSYMCHKSYCYRYRCAARSQNTPDSSASNLGFRCAAD	
Dasypus novemcinctus		VKKGGSYMCHKSYCYRYRCAARSQNTPDSSASNLGFRCAAN	
